# Satellite Derived Forest Phenology and Its Relation with Nephropathia Epidemica in Belgium

**DOI:** 10.3390/ijerph7062486

**Published:** 2010-06-09

**Authors:** José Miguel Barrios, Willem W. Verstraeten, Piet Maes, Jan Clement, Jean-Marie Aerts, Sara Amirpour Haredasht, Julie Wambacq, Katrien Lagrou, Geneviève Ducoffre, Marc Van Ranst, Daniel Berckmans, Pol Coppin

**Affiliations:** 1 Biosystems Department, M3-BIORES, Katholieke Universiteit Leuven, Willem de Croylaan 34, B-3001 Leuven, Belgium; E-Mails: willem.verstraeten@biw.kuleuven.be (W.W.V.); jean-marie.aerts@biw.kuleuven.be (J.-M.A.); sara.amirpourharedasht@biw.kuleuven.be (S.A.H.); daniel.berckmans@biw.kuleuven.be (D.B.); pol.coppin@biw.kuleuven.be (P.C.); 2 Laboratory of Clinical Virology, Hantavirus Reference Center, Rega Institute, Katholieke Universiteit Leuven, Minderbroederstraat 10, B-3000 Leuven, Belgium; E-Mails: piet.3.maes@uz.kuleuven.ac.be (P.M.); jan.clement@uz.kuleuven.ac.be (J.C.); julie.wambacq@biw.kuleuven.be (J.W.); Marc.Vanranst@uz.kuleuven.ac.be (M.V.R.); 3 Department of Experimental Laboratory Medicine, Katholieke Universiteit Leuven, Herestraat 49, B-3000 Leuven, Belgium; E-Mail: katrien.lagrou@uz.kuleuven.ac.be; 4 Scientific Institute of Public Health, Epidemiology, Juliette Wytsmanstraat 14, B-1050 Brussels, Belgium; E-Mail: gducoffre@iph.fgov.be

**Keywords:** nephropathia epidemica, hantavirus, forest phenology, remote sensing, bank vole

## Abstract

The connection between nephropathia epidemica (NE) and vegetation dynamics has been emphasized in recent studies. Changing climate has been suggested as a triggering factor of recently observed epidemiologic peaks in reported NE cases. We have investigated whether there is a connection between the NE occurrence pattern in Belgium and specific trends in remotely sensed phenology parameters of broad-leaved forests. The analysis of time series of the MODIS Enhanced Vegetation Index revealed that changes in forest phenology, considered in literature as an effect of climate change, may affect the mechanics of NE transmission.

## 1. Introduction

In Europe, nephropathia epidemica (NE) is a zoonotic disease caused by Puumala virus (PUUV). The main role in the virus transmission mechanism is played by a common arvicoline rodent species, the red bank vole (*Myodes glareolus*), that is native in Western Europe forests and acts as the virus reservoir [1]. The favourable habitat for bank voles in Belgium is broad-leaved forests (BLF) where illumination, soil and humidity conditions give rise to the existence of a moderately dense understory vegetation layer [2,3]. Similar to other forest fauna species, the bank vole population depends on interannual as well as on long term variations in the habitat conditions. Consequently, understanding and monitoring key factors associated to changes in the bank vole habitat is crucial to assess future implications of environmental changes for public health. Specially when, as cited by Clement *et al.* [4], different studies have shown the positive correlation between rodent density and prevalence of hantavirus infection.

One of the clearest evidences of the impact forest ecosystem variations can have in bank vole populations, and thus in human PUUV infections, is the mast phenomenon. The mast phenomenon is the abnormal abundant production of acorns, nuts and seeds by some tree species in certain years, also known as mast years. Several studies have demonstrated the impact of the mast phenomenon on rodent populations [5–8]. As for hantavirus infections in Belgium, recent studies [4,9,10] clearly show how masting can be connected to the observed temporal pattern of NE. The fact that several dominant tree species in Belgian BLF, like oaks (*Quercus* spp.) and beech trees (*Fagus sylvatica*), are known to have mast years, underlines the importance of tracking spatio-temporal patterns in vegetation dynamics.

Another key influencing parameter that determines rodent populations is the length of the forest growing season [11] since it affects the number of breeding events. Changes in the length of the growing season have been mentioned as one of the possible effects of climate change affecting the primary productivity of plants [12,13].

These vegetation-related phenomena and their link with epidemiologic pattern of NE highlight the need of exhaustive vegetation monitoring techniques. In this respect, remote sensing (RS) can offer valuable techniques and data sources to expand the knowledge on the linkages between spatio-temporal aspects of vegetation and disease occurrence. Various studies have confirmed the potential of RS in epidemiologic analyses [14–19].

In recent years valuable scientific contributions have increased our understanding of different aspects of the multiple interactions that determine the occurrence of NE in Belgium [3,4,9,10,20] as well as in other West European countries [4,21,22]. Undoubtedly, the interest in NE in Belgium has grown as consequence of the increased number of reported cases in recent years as it has been recorded by the Belgian Institute of Public Health [23]. The analysis of disease cases reports indicate that the incidence of NE is not uniformly distributed throughout the Belgian territory. Wallonia—southern Belgium—is clearly the region where most of the cases are reported. Based on the CORINE2000 Land Cover map (CLC2000) [24], we estimated that this region encompasses 84% of Belgian broad-leaved forests and 88% of Belgian mixed forests.

The analysis of the annual reports on NE cases in Belgium shows that the occurrence of peaks follows more or less regular intervals which can be associated to mast years, as pointed out by Tersago *et al.* [9] and Clement *et al.* [10]. These authors found that NE outbreaks show a positive correlation with autumn temperatures and summer temperatures one and two years before the peak, respectively.

Although the ties between vegetation and NE in Belgium and other West-European countries have been emphasized repeteadly in recent literature [3,4,9,10,20], little research has been conducted towards the exploration of RS techniques as a source of information on the dynamics of vegetation. In this study, the increasing availability (in time and space) of RS data sources is used to assess whether time series of satellite derived vegetation indices (VI) could provide valuable insights on the connections between vegetation dynamics and the epidemiological pattern observed in recent years in Belgium. Specifically, the objective of this study is to assess whether there is a relation between phenological parameters of BLF, as derived from spaceborne time series of vegetation, and the observed temporal NE pattern. The analysis is based on spaceborne data acquired by the MODIS sensor on board the Terra satellite for the period 2000–2008 and was conducted on 10 major forested areas in southern Belgium.

## 2. Methods

### 2.1. Study Area

**Figure 1.**
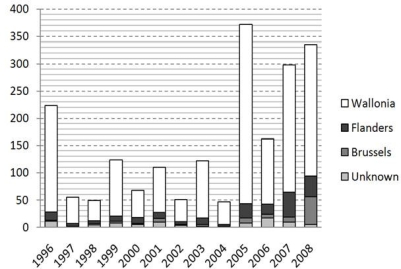
Number of NE reported cases in the different Belgian regions for the period 1996–2008 [24].

As stated earlier, recent reports [23,25] reveal that the number of NE cases has increased substantially in recent years and that this increase has been especially localized in Wallonia and the French Ardennes. Figure 1 illustrates this evolution for the different Belgian regions and published data from the early nineties reported the existence of an epidemic focus on both sides of the Franco-Belgian border [26].

**Figure 2.**
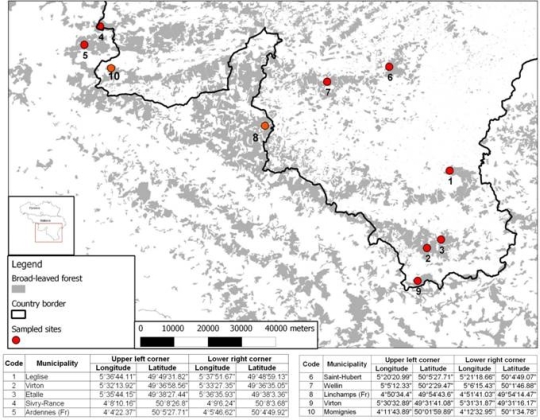
Sampled BLF areas in Belgium and France.

Based on these observations, this study focuses on forested areas in Wallonia (southern Belgium) and two sites in France where a high NE incidence has been observed [25]. The exact location of our sample sites is shown in Figure 2.

**Figure 3.**
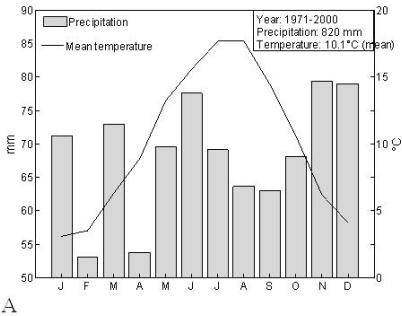

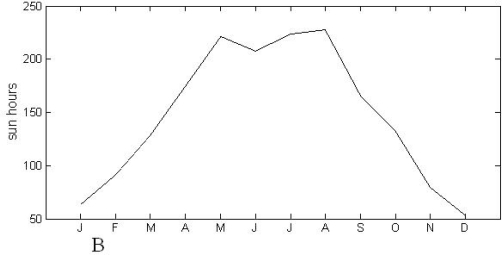
Climatogram (A) and Monthly average sun hours (B) in Belgium based on meteorological data for the period 1971–2000 [28].

The climate in the study area is, according to Köpper-Geiger climate classification, temperate, with warm summer and without dry season (denoted as Dfb) [27]. Average climatic data for Belgium [28], presented in Figure 3, confirm this description. The charts in Figure 3 show the unimodal pattern in temperature and sun hours which are decisive factors in shaping the reproductive cycle of plants in temperate regions [29].

### 2.2. MODIS EVI Time Series

The source of VI data is the MODIS (MODerate resolution Imaging Spectroradiometer) sensor on board the TERRA satellite. More specific, the dataset used in this study corresponds to the ‘Surface Reflectance 8-Day L3 Global 500 m’ product (MOD09A1). These data are freely available at the website of Earth Resources Observation Center (EROS) from the United States Geological Service (USGS) (http://glovis.usgs.gov/). The acquired dataset comprised imagery delivered every eight days at 500 m spatial resolution as a gridded product in the sinusoidal projection (SIN) for the period 2000–2008. The reflectance values offered in MOD09A1 are the result of the application of a number of algorithms intended to correct for the several effects (gaseous and aerosol scattering and absorption, adjacency effects, Bidirectional Reflectance Distribution Function and atmosphere coupling effects and contamination by thin cirrus) in order to estimate the spectral reflectances as if they were measured at ground level [30].

VI’s are commonly based on the contrast between red and infrared reflected energy observed in vegetative systems: green plants absorb red light and reflect infrared light. One of the most widely used VI is the Normalized Difference Vegetation Index (NDVI) which has been obtained from space at global scale for more than 25 years [31]. Although NDVI has extensively been used for a broad range of applications, it is subjected to some shortcomings such as canopy background contamination and signal saturation problems [31]. These shortcomings have encouraged research towards other VI’s [32] such as the Enhanced Vegetation Index (EVI). The EVI algorithm emerged as a response to NDVI sensitivity for canopy background and atmospheric influences [31] and uses the blue band to remove residual atmosphere effects caused by smoke and sub-pixel thin clouds [33]. This VI was considered suitable in our application, given the high cloudiness in the region of interest. The EVI was calculated as follows:

(1) $$\text{EVI}=2.\frac{\left(\rho_{\text{nir}}-\rho_{\text{red}}\right)}{L+\rho_{\text{nir}}+C_{1}\rho_{\text{red}}+C_{2}\rho_{\text{blue}}}$$

where ρ is reflectance; L a canopy background adjustment term; and C1 and C2, weigh the use of blue channel in aerosol correction of the red channel [31]. The ρred, ρnir and ρblue correspond in MODIS to bands 1 (620–670 nm), 2 (841–876 nm) and 3(459–479 nm), respectively.

#### 2.2.1. Extracting EVI values

EVI values were extracted from the MODIS dataset for pixels in 10 rectangular shaped locations covered by BLF. The location and extension of BLF patches was based on the Carte Numérique d'Occupation du Sol en Wallonie (COSW) [34] and on the CLC2000 [24] for the sites in Wallonia and in France, respectively.

The reflectance values used in the analysis were tested on quality by using the layer delivered in the MOD09A1 product related to band specific quality. Pixels non-compliant with a quality level catalogued as “Highest Quality” were discarded. EVI was computed when valid values for bands 1, 2 and 3 were found for the same date.

#### 2.2.2. EVI time series and phenological parameters

The computed EVI values were standardized to an arbitrarily defined fixed time step of 15 days by averaging, for each sampled site, the valid measurements registered for that lapse of time. This resulted in an EVI time series for the period 2001–2008 with 25 values per year.

The EVI time series were fitted to smooth curves from which a number of phenological parameters could be estimated. This was based on the procedures proposed by Jönsson and Eklundh [35] and incorporated in the TIMESAT software.

EVI time series were fitted to an asymmetric gaussian function. Prior to the actual fitting, the TIMESAT algorithm pre-processes the data in order to reduce the effect of noise in the signal. This step is based on the assumption that noise effect in vegetation indexes will cause especially low spikes in the signal.

Since this study is based on remotely sensed data, we define the vegetation growing season in terms of the energy reflectance in the red and infrared segment of the electromagnetic spectrum. Therefore, and for the case of BLF in temperate regions, the growing season can be conceived as the period of the year between green-up in spring when VI values start to increase, and senescence in fall when VI values approach a value of zero, *i.e*., growing season is the annual period of photosynthetical activity.

Based on the fitted function, TIMESAT computed a series of annual phenological parameters such as the start and end of the growing season, length of the growing season (LGS), rate of increase and decrease at the start and end of the season, respectively; seasonal amplitude and seasonal integrals [35].

The patterns of these parameters were examined for exploring possible connections between vegetation dynamics and NE incidence.

## 3. Results

The geographic coordinates of the sampled sites delimited an area equivalent to 97 pixels, representing more than 2,000 hectares of BLF. The sampled sites vary in terms of forest density and composition as well as in their spatial relation with other land cover classes. These elements exerted a great influence in the computed EVI values and partially explain the differences among sites. Despite the heterogeneity of sites, the derivation of phenological information revealed the existence of general phenological patterns in BLF which probably impacted the observed NE number of cases.

**Figure 4.**
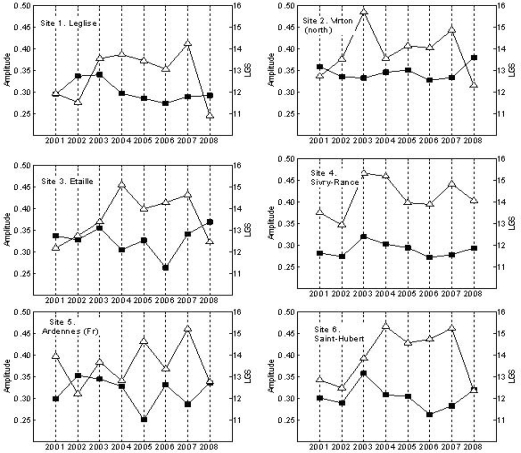

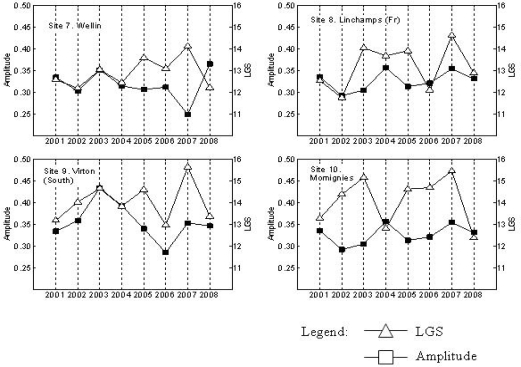
Length of growing season (LGS)—in periods of 15 days- and amplitude of EVI signal for the period 2001–2008 in 10 BLF areas in Belgium and France.

Annual VI’s of forested areas in temperate regions can be represented as unimodal functions with a maximum value in the summer after a monotonous increase in spring and followed by a monotonous decrease towards a minimum value in the winter. Such functions are shaped by the amplitude and phase which are strongly related, in the case of VI, to the difference between the maximum value and the base value, and to the LGS, respectively. The function is not necessarily symmetrical, which means that the maximum EVI value is not always reached at the middle of the growing season. The LGS and amplitude values are presented in Figure 4.

One of the most remarkable observed phenomena was the gradual increase in LGS in the period 2001–2007. Fitting these values to a linear function produced positive slope values for all sites, as can be seen in Table 1. The hypothesis that the slope value was statistically equal to zero was tested and, due to the difference in slope steepness and reduced number of years in the analysis, the hypothesis could not everywhere be rejected at a significance level of 0.05.

Nevertheless, a clear increasing trend in the LGS is observed and site specific conditions may explain the differences in magnitude. The satellite data of the Sivry-Rance, for instance, are strongly influenced by important agriculture areas. This explains that this and other sites showed a mild increase in LSG during the considered period. The increasing trend in LSG was more evident in highly dense forest complexes like the forests in Saint-Hubert, the French Ardennes, Leglise or Wellin.

**Figure 5.**
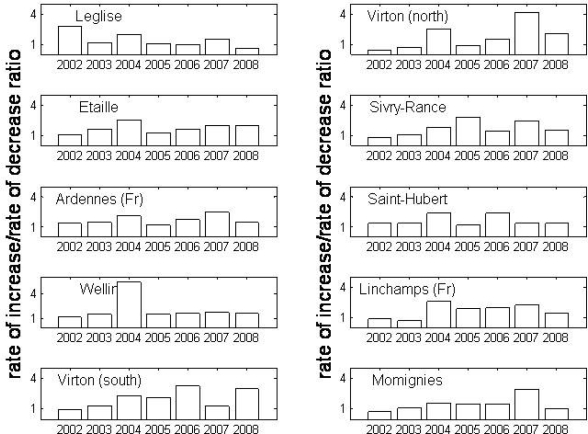
Annual rate of increase/rate of decrease ratio for the period 2002–2008 for 10 BLF sites in Belgium and France.

Complementary to the gradual increase in LGS, it is observed from the derived phenological data that the year preceding the highest NE peaks (2005, 2008 and locally, 2007) manifested a rapid green-up process together with a slow senescence, *i.e*., the annual EVI signal is asymmetrical with a longer period after the EVI peak has been reached. This can be derived from the ratio increase rate/decrease rate illustrated in Figure 5.

**Figure 6.**
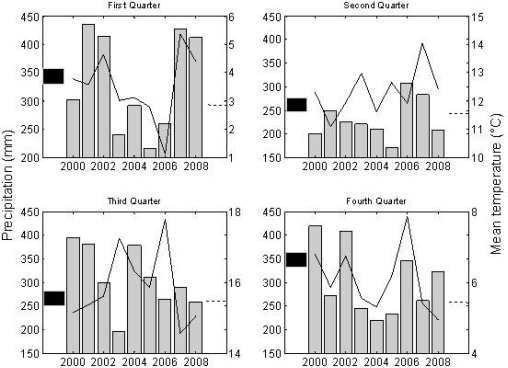
Average temperature (°C) (continuous line) and precipitation (gray bars) per quarter in the period 2000–2008 in the meteorological stations Forges (Chimay). Averages were estimated from records for the period 1980–2008: Mean temperature is the interrupted line; mean precipitation is the black bar.

Figure 4 shows also the amplitude values obtained from the EVI signal. For almost all sites, an increase in the amplitude value was observed in the year 2003. This is the effect of a low base value (average of the minimum values to the left and right of the annual EVI peak) caused by rainfall deficit. As shown in Figure 6, the year 2003 was characterized by temperatures above the average combined with rainfall deficit. The heat conditions of 2003 and 2006 have been proposed as the triggering factor of abundant seed production in the subsequent year and, therefore, explaining also the NE peaks of 2005 and 2008, respectively [9,10]. Another remarkable aspect of the amplitude lines is an incipient increasing trend from the year 2006 onwards. Although the dataset is not yet large enough to statistically confirm this trend, it appears to be a response to warmer conditions in combinations with non-dry years. Interestingly, this increase in amplitude occurs when high seed production are reported to occur in two consecutive years (2006 for beech, 2007 for oak), as presented in Table 2.

## 4. Discussion

Most of the discussion about vegetation dynamics and NE incidence is focused on the mast phenomenom and other aspects of vegetation dynamics have been underestimated. Our results and the extensive literature on phenological changes due to climate change suggest the need of studying vegetation dynamics as related to NE incidence from a broader perspective. The increase in frequency of the mast phenomenon in recent years (see for instance the years 2006 and 2007 in Table 2) can probably be situated in more encompassing trends of phenological changes taking place in vegetative systems.

Previous studies have already stressed the importance of vegetation phenology in rodents population. Stenseth *et al.*, for instance, succesfully tested the hypothesis of the seasonal length as an explanatory variable of cyclicity in gray red-backed vole (*Myodes rufocanus*) populations [36]. Klemola *et al.* proposed the onset and duration of the growing season of plants as determining factors of reproduction in rodents [11]. Knowing the importance of voles demography in NE [2], it is not surprising that the recent increase of NE reported cases has taken place in a context of gradual increase in LGS. An extended growing period will not only increase the number of breeding ocassions for rodents [11], but can enhance ecosystems carrying capacity. Our findings are coincident with other studies on phenology in which an enlargement of the growing period has been reported together with an early onset of photosynthetic activity [29,37]. These phenological trends have been attributed to global climate change and, in the case of the early spring onset, to increase in temperatures during that period [21,29,37].

Given the fact that we rely only on MODIS-derived data, statistical analysis on annual parameters do not have enough degrees of freedom to support conclusions at a high level of significance. Yet, evidence in literature of linkage between climate and onset and length of growing season showed a good correlation with our data as well. The correlation coefficients in Table 3 show that the temperature in the second quarter of the year and the date of start of the growing season tend to be negatively related; *i.e*., the warmer it is in spring the earlier the growing season starts. Furthermore, the figures in Table 3 suggest that the LGS is positively correlated with the temperature in June. Remarkable low correlation values were detected where the adjacency effects of neighboring land cover classes is expected to be the greatest: site 4.

The high ratio increase/decrease of the growing period appeared to influence the NE mechanics given that the highest values occured in the growing period preceding the highest NE peaks. Those years were also characterized by high tree seed production. This suggests that the rodent population and the interactions amongst individual rodents are impacted by at least two factors: the crop production abundance of beech and oak, and the favourable and long conditions to mate. This combined effect might explain outstanding NE peaks in 2005 and 2008 in contrast to other mast years resulting in less outspoken NE peaks.

Longer periods of milder weather conditions and greenness in the forest as well as positive changes in forest growth as consequence of climate change may also be an important aspect attracting humans to forest [38], thereby increasing exposure risk. The most important factor leading to NE is without doubt the degree of human exposure to aerosolized excreta of bank voles. Although this exposure does not occur exclusively in forested areas, outdoor activities in vole infested environments may imply a great risk. This can be exemplified with the first NE outbreak documented in Germany, with 24 cases within two weeks during a winter bivouac of the U.S. Army on a vole-infested terrain near Ulm (S.-Germany) in January 1990. During the same winter period, not a single civilian case of NE was registered in the whole same region [39]. Works cited by Clement *et al.* [40] refer also to the risk of infection campers and trekkers face when sleeping among rodent burrows or in confined places infested with rodents like caravans, mountain refuges, hunting lodges, fishing cabins, *etc*.

## 5. Conclusions

The increasing hantavirus incidence in recent years has been associated to global scale climate changes [3,4,10] influencing the dynamics of forests and thereby inducing changes in the habitat of bank voles. Recent literature showed that one of the major impacts of a changing environment in forests is its effect in phenological features [29,37]. Based on our results we can conclude VI time series can provide valuable information, in the form of derived phenological parameters, that can lead to the identification of favourable conditions for NE outbreaks. This exploratory work confirms also that the relative young history of records of epidemic increase of NE as well as the short existence of the MODIS sensor obstructs the statistical reliability of annual parameters. On the other hand, the spatial dimension of satellite data and its temporal resolution gives an added value to RS techniques (as compared to climatic data) since it can be used to delineate or model the geographical extent of phenological patterns.

## Figures and Tables

**Table 1.** Slope and p statistic for linear fitting of LGS values for the period 2001–2007.

**Site**	**Slope**	**p**	**Site**	**Slope**	**p**

1. Leglise	0.3525	0.04324*	6. Saint-Hubert	0.4432	0.0162*
2. Virton (North)	0.2089	0.29213	7. Wellin	0.2496	0.0332*
3. Etaille	0.3943	0.02538*	8. Momignies	0.2375	0.2218
4. Sivry-Rance	0.1604	0.39400	9. Virton (South)	0.1871	0.3277
5. Ardennes (Fr)	0.2532	0.21977	10. Linchamps (Fr)	0.2496	0.27595

* Hypothesis of slope = 0 rejected when p < 0.05.

**Table 2.** General ratings of fruit production in forests. Source: Le Comptoir Forestier/Région Wallonne.

Year		1995	1996	1997	1998	1999	2000	2001	2002	2003	2004	2005	2006	2007	2008	2009
Rating	Beech	+++	o	o	++	o	+++	o	+++	o	+++	o	+++	+	o	+
	Native oak	+++	o	o	+++	o	+++	o	o	o	+++	o	+	+++	o	+

+++ = very good year;

++ = good year;

+ = moderate;

weak or absent production = o.

**Table 3.** Correlation coefficients (r) for the relations: A. Average temperature in the second quarter and onset date of the growing season, and; B. Average temperature in June and length of the increase phase for the different sites (* = p < 0.05).

**Site**	**A**		**B**		**Site**	**A**		**B**	
	
	**r**	**p**	**r**	**p**		**r**	**p**	**r**	**p**

**1**	−0.628	0.0952	0.729	0.0400*	6	−0.364	0.3749	0.784	0.0214*
**2**	−0.483	0.2250	0.732	0.0391*	7	−0.786	0.0206*	0.669	0.0696
**3**	−0.266	0.5243	0.657	0.0768	8	−0.597	0.1185	0.709	0.0487*
**4**	−0.382	0.3501	0.489	0.2183	9	−0.692	0.0569	0.669	0.0696
**5**	−0.811	0.0147*	0.760	0.0285*	10	−0.179	0.6710	0.726	0.0416*
